# Solar Retinopathy: A Multimodal Analysis

**DOI:** 10.1155/2013/906920

**Published:** 2013-02-12

**Authors:** Claudia Bruè, Cesare Mariotti, Edoardo De Franco, Yale Fisher, Jacopo Maria Guidotti, Alfonso Giovannini

**Affiliations:** ^1^Ophthalmology Section, Department of Neuroscience, Polytechnic University of Marche, Via Brecce Bianche, 60121 Ancona, Italy; ^2^Vitreous-Retina-Macula Consultants of New York, 460 Park Avenue, 5th Floor, New York, NY 10022, USA

## Abstract

*Purpose*. Solar retinopathy is a rare clinical disturbance, for which spectral-domain optical coherence tomography (SD-OCT) findings are not always consistent. We report on two cases of solar retinopathy and discuss its differential diagnosis. *Methods*. This is an observational case study. *Results*. A 12-year-old female was referred to ophthalmology for bilateral scotoma. Visual acuity was 20/50 in both eyes. Fundus examination was unremarkable, except for slight yellowish material in the central macula, bilaterally. SD-OCT revealed juxtafoveal microcystic cavities in the outer retina, interruption of the external limiting membrane and the inner and outer segment junctions, with disorganized material in the vitelliform space. Fundus autofluorescence showed hypoautofluorescence surrounded by a relatively hyperautofluorescent ring, bilaterally. Similar clinical and morphological findings were detected in a 27-year-old male. *Conclusions*. Solar retinopathy has a subtle presentation and patients often deny sun-gazing. SD-OCT and fundus autofluorescence are noninvasive and useful tools for its diagnosis.

## 1. Introduction

Solar retinopathy is a rare ocular lesion that can result from unprotected solar eclipse viewing and also from minimal gazing at the sun. The consequent photochemical/thermal retinal damage [1] often has a subtle presentation, which can be misleading for its diagnosis.

Spectral-domain optical coherence tomography (SD-OCT) is a noninvasive imaging technique that is useful for the detection of foveal impairment and to outline the location and extension of retinal injury from acute solar retinopathy [2]. However, acute solar retinopathy can sometimes share biomicroscopic and SD-OCT foveal findings with pathologies such as whiplash injury, sunbed exposure, ocular trauma, the initial stages of an idiopathic macular hole, persistent retinal defects following successful macular hole repair, idiopathic parafoveal telangiectasis, and solitary macular cysts [3, 4]. Differential diagnosis is often handled according to patient history combined with OCT findings.

Fundus autofluorescence (FAF) is a relatively new and noninvasive technique that is based on the autofluorescent properties of retinal fluorophores, such as lipofuscin, which is mainly located in the retinal pigment epithelium (RPE). Reduced content of lipofuscin in the RPE has been described in cases of light-induced loss of photoreceptors [5]. A history of acute solar retinopathy in some cases is not reliable and fluorescein angiography does not provide further information. Instead, SD-OCT provides a diagnostic aid for relevant findings, even if these are common to several retinal diseases. Furthermore, FAF represents an effective tool to detect subtle changes in the RPE and to provide a better understanding of the pathophysiology of solar retinopathy.

We report here on the clinical, SD-OCT and FAF imaging findings from four eyes of two subjects with mild RPE damage following unprotected sun-gazing.

## 2. Case 1

**Figure 1:**
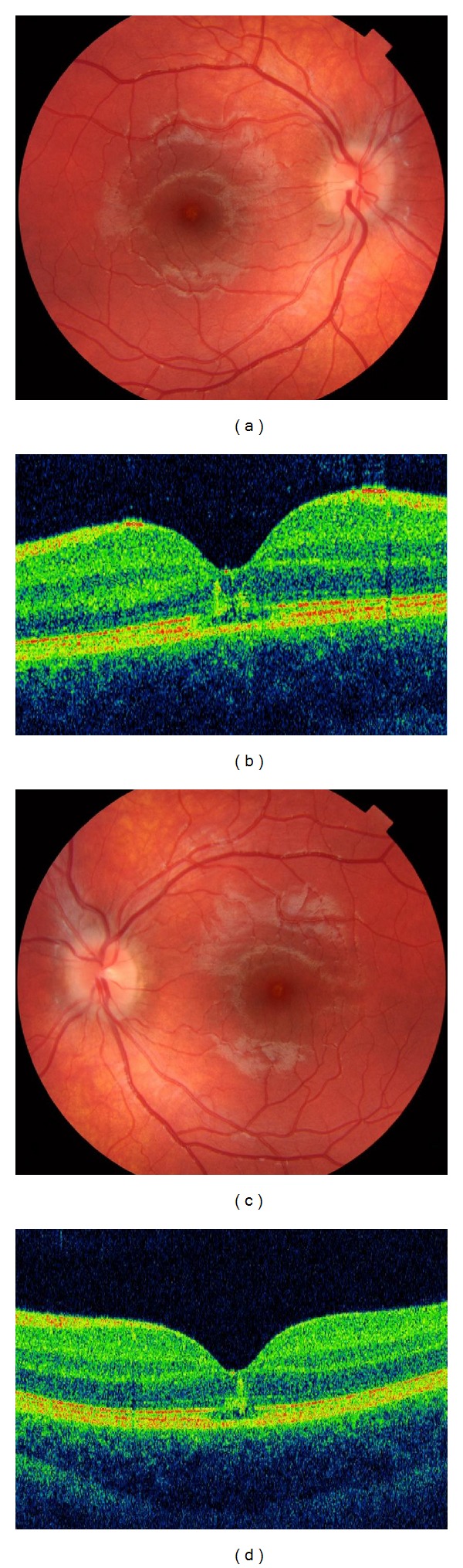
Ophthalmoscopy of the right and left eye of Case 1, showing a normal macula with a slight reduction of the foveal reflex (a, c). SD-OCT showed a maintained foveal contour, juxtafoveal microcystic cavities in the outer retina, increased foveal rod-shaped full-thickness hyperreflectivity that extended from the outer segments of the photoreceptors and RPE to the inner layer of the retina, and a slight interruption of the external limiting membrane and of the inner and outer segment (IS/OS) junctions, with disorganized material in the “vitelliform space” (b, d).

A 12-year-old Caucasian female with blond hair and blue eyes complained about a sudden bilateral blurring in her central visual field a few hours after a long walk in the mountains on a sunny day. The patient denied any direct sun-gazing, wearing of a hat and sunglasses, use of systemic and topical drugs, and any previous medical and ocular history. Her visual acuity was 20/50 in both eyes. Anterior segment examination was unremarkable in each eye. On dilated fundus examination, the foveal reflex was reduced and small yellowish juxtafoveal lesions were observed (Figures 1(a) and 1(c)). SD-OCT using a (Topcon America, Paramus, NJ, USA) showed a maintained foveal contour, juxtafoveal microcystic cavities in the outer retina, increased foveal rod-shaped full-thickness hyperreflectivity that extended from the outer segments of the photoreceptors and RPE to the inner layer of the retina, and a slight interruption of the external limiting membrane and of the inner and outer segment (IS/OS) junctions, with disorganized material in the “vitelliform space” (Figures 1(b) and 1(d)). Fundus autofluorescence (FAF) showed a well-demarcated hypoautofluorescence that corresponded to the outer retina/RPE defect and was surrounded by a slightly hyperautofluorescent ring, bilaterally. High-dose steroid therapy was started. At 4 months of followup, her visual acuity improved to 20/20.

## 3. Case 2

**Figure 2:**
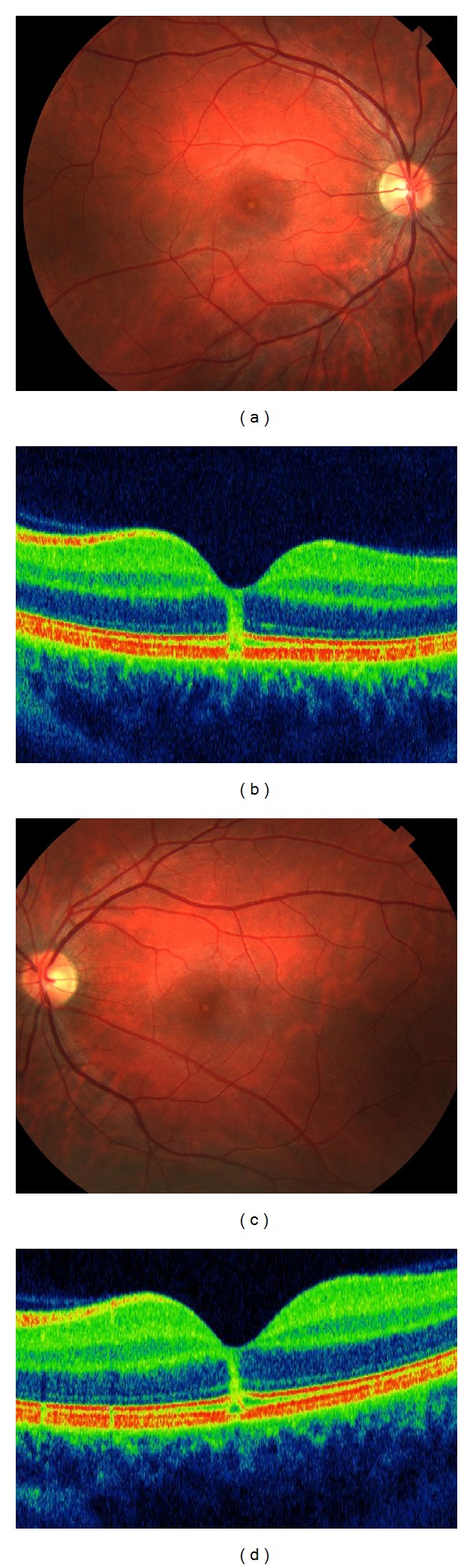
The color fundus of the right and left eyes showed a reduced foveal reflex (a, c). SD-OCT detected increased foveal rod-shaped full-thickness hyperreflectivity that extended from the outer segments of the photoreceptors and the RPE to the inner layer of the retina and interruption of the external limiting membrane and of the IS/OS junction, bilaterally (b, d).

A 27-year-old Caucasian male who worked in the Navy came for a medical retinal consultation following blurry vision in both eyes. He reported looking at the sun for few minutes one day prior to the presentation, without sunglasses and without wearing a hat. His visual acuity was 20/100 in both eyes. Anterior segment examination was normal. Intraocular pressure was normal. On dilated fundus examination, the foveal reflex was abnormal and RPE changes were detected at the fovea (Figures 2(a) and 2(c)). SD-OCT showed a maintained foveal contour, absence of vitreoretinal traction, increased foveal rod-shaped full-thickness hyperreflectivity that extended from the outer segments of the photoreceptors and the RPE to the inner layer of the retina, and interruption of the external limiting membrane and of the IS/OS junction, bilaterally (Figures 2(b) and 2(d)). FAF demonstrated a foveal hypoautofluorescent area that was circled by a slightly hyperreflective ring, bilaterally. The patient was immediately started on oral prednisone (1 mg/kg). One week later, his visual acuity was markedly improved to 20/40, but the subject was still complaining of central scotoma and metamorphopsia. Further fundus examination showed a reduction in the yellow spot. At SD-OCT, the hyperreflective band had receded. At 6 months, the patient showed complete recovery to 20/20 visual acuity. SD-OCT demonstrated that the external limiting membrane and the IS/OS junction were intact. The FAF findings were isoautofluorescent.

## 4. Discussion

Solar retinopathy is a maculopathy that arises following direct exposure to solar radiation. There is an extensive spectrum of solar-induced lesions and wide individual variability in the responsiveness and the acquisition of these injuries. Several conditions can influence the severity of the clinical and morphological presentation: intensity, duration, and light spectrum of the exposure; clearness of the ocular media; ocular pigmentation; body temperature; environmental conditions, such as highly reflective surroundings and atmospheric changes [1].

Solar retinopathy is usually seen as bilateral, even if unilateral and asymmetric manifestations have been described [2, 6]. Fundus examinations in acute phases can show a small yellow spot at the fovea, encircled by faint gray granular pigmentation [3]. The yellowish discoloration will usually become faint with time, leaving a pathognonomic reddish spot [3]. Rai et al. [7] reported that only 51% of subjects affected by solar retinopathy referred to a sun-gazing history. These patients represent more of a diagnostic challenge. Case 1 in our series denied any sun-gazing, and she referred only to outdoor walking in the mountains on a sunny day a few hours prior to the ocular complaint. Her ophthalmoscopic examination might have led to the diagnosis of a pseudovitelliform lesion. Indeed, SD-OCT showed disorganized material at the level of the layer between the RPE and the photoreceptor IS/OS interface. Here, FAF was effective to settle the diagnosis, as it showed hypoautofluorescence that corresponded to a defect of the IS/OS junction. This was encircled by a faint hyperautofluorescent ring, which ruled out pseudovitelliform dystrophy. Well-demarcated areas of hypoautofluorescence that correlate to loss of the IS/OS junction have been described, although this was hypothesized to be due to RPE cell loss [8]. However, none of the eyes of our patients showed atrophy of the RPE; instead, they showed a slight reduction of the reflectivity that was documented by SD-OCT. Indeed, the hypoautofluorescence detected was more probably due to a loss of photoreceptors following injury to the RPE cells.

Solar radiation is absorbed directly from the RPE. This damage is responsible for the reduced lipofuscin content of the RPE cells, which follows from the reduced phagocytosis of photoreceptor outer segments due to a disruption of the outer segments-to-RPE interdigitation [9]. Although a hyperautofluorescent ring surrounding the reduced central autofluorescence has been described once previously [8], to the best of our knowledge, our study is the first to provide a reasonable explanation for this finding. The damage to the visual photo pigments or the sensitive cones by solar radiation induces a lack of normal outer segment processing [9], which can lead to increased accumulation of outer segments in the outer retina and subretinal space, as was revealed by SD-OCT in both of our cases. This material is a precursor of the bisretinoid N-retinylidene-N-retinylethanolamine (A2E) [10] in the outer retina, which is visible as an increasingly thick layer at that level, and it is yellow and autofluorescent. Indeed, FAF imaging is particularly useful to identify unphagocytosed photoreceptor outer segments [11]. Moreover, 25% of the macular pigment is localized in the outer segments of the photoreceptors [12], the loss of which in solar retinopathy can increase the autofluorescence detected with FAF examination. The outer retinal hole detected by SD-OCT in Case 1, with the accompanying inner retinal cysts and disruption of the IS/OS junction, are the same findings as those in patients affected by an early stage of MacTel type 2 [13]. To rule out this progressive disease, the detection of the diffuse area of increased autofluorescence at FAF was helpful, rather than the invasive fluorescein angiography, or the detection of the right angle vessel diving down into the outer retina in the temporal fovea.

Gass postulated that whiplash-like injuries can present similar ophthalmoscopic findings to solar retinopathy [3]. Diriment to the solar retinopathy findings, SD-OCT shows a hyperreflective line on the inner retinal surface. An outer retinal hole is a rare manifestation in Stargardt's disease [14], and it would show rounded edges and be progressive, while in solar retinopathy, a hole would have squared sides and be stationary. Early vitreomacular traction was described by Johnson [15], and it can produce inner and outer retinal cystic changes at the fovea that will rarely result in a rectangular outer retinal hole. In such cases, SD-OCT reveals the epiretinal membrane. Tamoxifen retinopathy can also show a rectangular outer retinal cyst with cystoid macular edema [16]. The relevance here would be a history of tamoxifen use for several years and the presence of white crystals in the inner retina.

Considering the increased foveal rod-shaped full-thickness reflectivity in both of our cases, this was seen the day after the solar radiation exposure, and we can consider this as a very early stage and severe form of solar maculopathy. As far as we are aware, this finding has been described only once before [17], in a case of a 14-year-old boy, as seen 10 days after unprotecting viewing of a solar eclipse.

The severity of the anatomical lesions that we detected by SD-OCT might be explained by the concomitance of several risk factors in each of our patients. Case 1 showed emmetropia, with reduced ocular pigmentation. Here, the long walk on a sunny day will have increased her body temperature and induced a mydriatic effect. Along with the high altitude of the mountain, these are all conditions that predispose to higher exposure to solar radiation [1]. Case 2 was working in the Navy along the sea coast with highly reflective surroundings [1]. SD-OCT findings in our cases were at a more precocious phase than previously described, and some recovery was detected at 7 days after the solar exposure and the treatment. The prompt response to treatment led us to hypothesize that the first pathological mechanism that occurs during this retinal injury is inflammation. The hyperreflective area involving the inner and outer retina at the fovea might represent inflammatory cells that might have arrived following chemokine upregulation in the traumatized retinal tissue, following the breakdown of the blood-retinal barrier. Photocoagulation would probably have occurred through a thermal mechanism, when the light produced coagulation of the retinal proteins [1], which appeared hyperreflective at SD-OCT.

Another pathophysiological aspect of solar retinopathy can be photochemical injury mediated by highly reactive free radicals [1], as postulated by Gass [3], through light-induceddamage of the apical melanosomes of the RPE by the blue-light wavelengths. As these pathological changes occur mainly at the outer retina, it is possible that our patients had a more severe form of solar retinopathy, with the involvement of the whole foveal retina, or that the dominant pathophysiological mechanism in our cases was thermal.

In conclusion, our cases suggest multifactorial pathogenesis of solar retinopathy, and they highlight the synergetic effectiveness of the use of SD-OCT and FAF to establish the diagnosis of solar retinopathy in its relatively subtle manifestations.
